# Cross-Task Consistency of Electroencephalography-Based Mental Workload Indicators: Comparisons Between Power Spectral Density and Task-Irrelevant Auditory Event-Related Potentials

**DOI:** 10.3389/fnins.2021.703139

**Published:** 2021-11-16

**Authors:** Yufeng Ke, Tao Jiang, Shuang Liu, Yong Cao, Xuejun Jiao, Jin Jiang, Dong Ming

**Affiliations:** ^1^Tianjin Key Laboratory of Brain Science and Neural Engineering, Tianjin International Joint Research Centre for Neural Engineering, Academy of Medical Engineering and Translational Medicine, Tianjin University, Tianjin, China; ^2^National Key Laboratory of Human Factors Engineering, China Astronaut Research and Training Centre, Beijing, China

**Keywords:** mental workload, EEG, task-irrelevant auditory event-related potentials, power spectral density, cross-task comparison

## Abstract

Mental workload (MWL) estimators based on ongoing electroencephalography (EEG) and event-related potentials (ERPs) have shown great potentials to build adaptive aiding systems for human–machine systems by estimating MWL in real time. However, extracting EEG features which are consistent in indicating MWL across different tasks is still one of the critical challenges. This study attempts to compare the cross-task consistency in indexing MWL variations between two commonly used EEG-based MWL indicators, power spectral density (PSD) of ongoing EEG and task-irrelevant auditory ERPs (tir-aERPs). The verbal N-back and the multi-attribute task battery (MATB), both with two difficulty levels, were employed in the experiment, along with task-irrelevant auditory probes. EEG was recorded from 17 subjects when they were performing the tasks. The tir-aERPs elicited by the auditory probes and the relative PSDs of ongoing EEG between two consecutive auditory probes were extracted and statistically analyzed to reveal the effects of MWL and task type. Discriminant analysis and support vector machine were employed to examine the generalization of tir-aERP and PSD features in indexing MWL variations across different tasks. The results showed that the amplitudes of tir-aERP components, N1, early P3a, late P3a, and the reorienting negativity, significantly decreased with the increasing MWL in both N-back and MATB. Task type had no obvious influence on the amplitudes and topological layout of the MWL-sensitive tir-aERP features. The relative PSDs in θ, α, and low β bands were also sensitive to MWL variations. However, the MWL-sensitive PSD features and their topological patterns were significantly affected by task type. The cross-task classification results based on tir-aERP features also significantly outperformed the PSD features. These results suggest that the tir-aERPs should be potentially more consistent MWL indicators across very different task types when compared to PSD. The current study may provide new insights to our understanding of the common and distinctive neuropsychological essences of MWL across different tasks.

## Introduction

Mental workload (MWL) has been stated as a multidimensional construct determined by the characteristics of the task (e.g., demands, performance), of the operator (e.g., skill, attention), and, to a certain degree, the environmental context in which the performance occurs (Young et al., 2015). Generally, MWL reflects task difficulty and the associated mental effort (Gevins and Smith, 2006). That is the reason why the MWL of the subjects was modulated by task difficulty in almost all neuroergonomic MWL studies. Previous studies have found that overload mental effort may be harmful to human performance and situation awareness (Wickens, 2008). In recent years, the objective estimation of MWL using neural signals has become an important topic in the field of human factors and neuroergonomics (Tao et al., 2019). Objectively real-time monitoring of MWL using neurophysiological metrics is essential to build closed-loop adaptive aiding systems for complex and safety-critical human–machine systems (Vidulich and Tsang, 2015; Teo et al., 2018, 2020). Although the neuroergonomic methods for MWL estimation have attracted much attention in the past years, the common neuropsychological essence of MWL across different tasks or mental activities is still to be uncovered.

The foundation of measuring MWL using neural signals like electroencephalography (EEG) is that different amounts of cognitive/neural resources may be engaged in different task difficulty levels. Over the past years, features from EEG have shown great potentials to estimate MWL in real time (Gevins and Smith, 2006; Haynes and Rees, 2006; Tao et al., 2019). Among them, the most extensively investigated features are the power spectral densities (PSDs) of ongoing EEG and the event-related potentials (ERPs). EEG power in the alpha band (α, 8–13 Hz) has been found to be negatively correlated with MWL in tasks such as working memory (Gevins et al., 1998; Pesonen et al., 2007), simulated driving (Lei and Roetting, 2011; Borghini et al., 2014; Yang et al., 2020), and multitasking (Ke et al., 2015a; Puma et al., 2018) in previous studies, possibly due to its link to arousal level, idling, cortical inhibition (Uusberg et al., 2013), and the default mode network activation (Knyazev et al., 2011). In recent years, EEG power features in frequencies from 0.5 to over 100 Hz have been found to be modulated by task difficulties and employed to estimate MWL with machine learning techniques in many different tasks (Brouwer et al., 2012; Wang et al., 2012; Borghini et al., 2014; Casson, 2014; Fallahi et al., 2016; Yin and Zhang, 2017; Kakkos et al., 2019; Tao et al., 2019; Zhang et al., 2019; Chakladar et al., 2020). The satisfactory performance of EEG-based MWL estimators trained and tested in the same task (within-task) have shown its potential for practical application. However, the cross-task application, in which MWL estimators are trained on one or a set of tasks and applied to other tasks, is still a challenge, although a few studies proposed some potential solutions (Haynes and Rees, 2006; Ke et al., 2014, 2015a; Dimitrakopoulos et al., 2017; Zhang et al., 2018; Zhao et al., 2018; Boring et al., 2020). The main reason for the cross-task challenge may lie in the difference of the neurophysiological responses between different task types (Baldwin and Penaranda, 2012; Ke et al., 2014, 2015a), that is, although a variation of MWL may cause the variation of EEG features in a certain task type, the differences between task types may lead to very different neurophysiological responses because different tasks use different cognitive strategies and thus occupy different neural resources. A significant main effect of task has been observed for spectral power, especially the alpha band, in previous cross-task studies (Baldwin and Penaranda, 2012; Ke et al., 2015a). It implies that considerable task-specific features in EEG spectrum have not been revealed and may impair the cross-task generalizability of the machine learning techniques. That should be the reason why poor performances have been obtained in cross-task MWL measurement studies which employed spectral features (Baldwin and Penaranda, 2012; Ke et al., 2014, 2015a).

The theory of cognitive resources and its relation to the generation of ERPs may provide new insights into the common neuropsychological essence and the more robust MWL-sensitive EEG features between different tasks. According to the theory of cognitive resources, limited capacity is the fundamental characteristic of human cognitive resources (Wickens, 2008). That means less residual cognitive capacity will be available for additional tasks or perceptual stimuli if you are engaged in a more demanding task. A substantial literature spanning over two decades has indicated that the magnitude of mental resources that are being recruited and the duration that these resources are being utilized to process a particular stimulus can be revealed by the amplitudes and the latencies of ERPs (Kok, 1997; Stewart and Paulus, 2013; Ghani et al., 2020a). From the aspects of mental resources theory, high-load mental processing may employ more attentional resources and reduce the capacity of the brain to recognize visual or auditory events (Wickens, 2008). As evidence for the mental resources theory, some studies found that ERP decreased in magnitude when sensory stimuli were presented in conjunction with the performance of other tasks (Isreal et al., 1980; Kida et al., 2004, 2012; Bonato et al., 2015; Ke et al., 2015b; Jaquess et al., 2017). Scheer et al. (2016) have found that steering demands on mental resources diminished the early P3, late P3, and re-orientation negativity (RON) components of the ERP of task-irrelevant environmental sounds. Studies on the phenomenon termed “inattentional deafness,” which refers to the neglect of unexpected auditory information, have also provided evidences for the weakened ERP responses to auditory stimuli under demanding situations (Giraudet et al., 2015; Causse et al., 2016; Scheer et al., 2018). In general, these literatures tend to show that less remaining resources may be left for processing unexpected sensory information, and thus weaker brain responses, like ERPs, to sensory stimuli may be observed when tasks involving a high cognitive load occupied the limited attentional capacities. In this context, the unfavorable side is that operators may fail to detect unexpected alerts in demanding situations. However, this phenomenon may also provide an alternative approach for MWL measurement.

Inspired by the above-mentioned findings, the amplitude of ERP components evoked by sensory stimuli, which can index the amount of attentional resources allocated to process the stimuli, has been employed to index MWL in previous studies in the past decades. Early studies on the relationship between task demand and ERPs can be traced back to the 1980s. It has been proven in dual-task studies that increases in the difficulty of a complex perceptual–motor primary task resulted in decreases in the amplitude of P300 elicited by secondary task tones which required occasional responses but increases in the amplitude of P300 elicited by the primary task (Wickens et al., 1983; Sirevaag et al., 1989). Table 1 shows the typical task-irrelevant auditory ERP (tir-aERP)-based MWL studies in the past decades. In these studies, nearly all the task-irrelevant probes were auditory because almost all the tasks highly depended on visual attention. Therefore, visual probes would be more intrusive to and compete visual resources with the primary tasks. A few studies which employed auditory oddball tasks requiring response to the deviants as the secondary task in recent years suggested that amplitudes of ERP components like P2 and P3 elicited by deviant tones decreased in high-difficulty tasks (Giraudet et al., 2016; Horat et al., 2016; Solis-Marcos and Kircher, 2019). However, the requirement to respond to secondary tasks from subjects would be unacceptable for safety-critical systems because it has been found to disrupt the performance of primary tasks (Kramer et al., 1995). As a less intrusive way, the auditory oddball paradigm without response has been employed, and lower amplitudes of N100, N200, P300, and MMN were found in high-MWL conditions (Kramer et al., 1995; Dehais et al., 2019). An alternative to the secondary task methods has been referred to as the task-irrelevant probes method, which presents auditory or visual stimuli accompanying the task of interest and does not require the subjects to respond or count (Papanicolaou and Johnstone, 1984). By measuring ERPs elicited by an ignored single-stimulus tone (Allison and Polich, 2008; Roy et al., 2016a; Ghani et al., 2020b) or variable-frequency tone sequence (Takeda et al., 2016) while the participants focus on the task of interest, the authors reported decreases in N1, N2, P2, and/or P3 component amplitudes with increases in MWL. By assuming that novel stimuli would be more effective in indexing MWL than simple tones, Miller et al. (2011) published a study using a variety of novel, task-irrelevant auditory stimuli and found that N1, P2, P3 and late positive potential (LPP) component amplitudes were inversely related to task difficulty. Dyke et al. (2015) found that complex auditory stimuli were significantly more effective in indexing cognitive workload than simple stimuli due to their ability to elicit the early P3a (eP3a) component, a robust orienting response which decreased monotonically as a function of MWL. Not only the eP3a but also the amplitudes of N1, P2, and lP3a and/or the RON elicited by the novel complex sounds have been claimed to be diminished by increased MWL in recent studies (Deeny et al., 2014; Dyke et al., 2015; Scheer et al., 2016; Shaw et al., 2018).

**TABLE 1:** Overview of the previous typical tir-aERP-based mental workload (MWL) studies.

Study	Primary task	Paradigm/stimulus	ERP measures
Giraudet et al. (2016)	Air traffic control	Oddball*/pure tones	P300 amplitude ↓
Horat et al. (2016)	Tone discrimination	Oddball*/pure tones	P2, P3a and P3b amplitudes ↓
Solis-Marcos and Kircher (2019)	Tracking task	Oddball*/pure tones	N1 latency ↑; P3 amplitude ↓
Kramer et al. (1995)	Radar monitoring	Oddball/pure tones	N100, N200, P300 and MMN amplitudes ↓
Dehais et al. (2019)	Flying simulation	Oddball/pure tones	P3 amplitude ↓
Ullsperger et al. (2001)	Gauge monitoring and mental arithmetic	Oddball/tones and novel sounds	N1 and P3 amplitudes ↓
Allison and Polich (2008)	“First person shooter” video game	Single stimulus/pure tone	P2, N2 and P3 amplitudes ↓
Roy et al. (2016a)	Multi-attribute Task battery	Single stimulus/pure tone	P2 latency ↓
Takeda et al. (2016)	Driving simulation	Variable tone frequency sequence	N1 and P2 amplitudes ↓
Ghani et al. (2020b)	Tilt-ball game	Single stimulus/pure tone	N1 amplitude ↓
Miller et al. (2011)	Tetris^®^ game	Novel complex sounds	N1, P2, P3 and LPP amplitudes ↓
Deeny et al. (2014)	Myoelectric control of a virtual limb	Novel complex sounds	P200, P300 and LPP amplitudes ↓
Dyke et al. (2015)	Tetris^®^ game	Novel/repeated simple/complex sounds	N1, eP3a, lP3a amplitudes ↓
Scheer et al. (2016)	Steering task	Beep tones, novel complex sounds	Early P3, late P3 and the RON amplitudes ↓
Shaw et al. (2018)	Stimuli detection	Novel complex sounds	Novelty P3 ↓

*, response to the auditory stimulus required in the task; ↓, the measure(s) decreased with an increase in the task difficulty or MWL; ↑, the measure(s) increased with an increase in the task difficulty or MWL.

Taken together, the amplitude of ERPs elicited by task-irrelevant auditory probes has the potential to index MWL variation for many different tasks as has been claimed in the above-mentioned studies. Furthermore, the novel complex sounds, as has been employed by Dyke et al. (2015), should be a better choice for the auditory probes. By integrating the above-mentioned findings and the mental resources theory, the assumptions for these tir-aERP-based MWL studies should be as follows: (i) the residual mental resources allocated to task-irrelevant probes are reduced if a higher proportion of the limited mental resources was involved in a demanding task (Kahneman, 1973; Wickens, 2008) and (ii) the amplitude variation of tir-aERPs elicited by task-irrelevant probes can reflect the amount of mental resources to these probes (Ghani et al., 2020a). These previous findings provide novel possibilities to extract MWL-sensitive features, which may be more generalizable across different task types, from ERPs.

The current study aimed to compare the consistency between ongoing EEG power and tir-aERPs in indexing MWL across different tasks. Task-irrelevant auditory probes were presented to the participants and ignored while they performed the tasks. The effects of MWL on the magnitude of tir-aERPs and ongoing EEG power were examined under two MWL levels (low and high) and compared between two task types [N-back and multi-attribute task battery (MATB) (Santiago-Espada et al., 2011)]. Based on the task-irrelevant characteristics and the consistent findings that MWL diminished the amplitudes of tir-aERPs in almost all previous studies, we expected that the amplitude of tir-aERPs should be consistently diminished by MWL in the two different tasks and less affected by task type. It was conjectured that the power spectrum of ongoing EEG should also respond to MWL variation, but it should be more sensitive to task type according to its task-relevant characteristics and the findings in previous studies. The performance of classification across the two tasks was also examined and compared between the ERP and PSD features. The main significance is that the current study firstly examined the effects of MWL and task type on the most investigated MWL-sensitive EEG features in one study design and may provide new insights to our understanding of the common and distinctive neuropsychological essences of MWL across different tasks and new methodology for future MWL estimation studies.

## Materials and Methods

### Participants

Seventeen healthy subjects (10 male and seven female, aged 20–26) with normal hearing and normal or corrected-to-normal vision voluntarily participated in this study with informed consents. This study was carried out in accordance with the recommendations of the institutional review board of Tianjin University. The study protocol was approved by the Ethics Committee of Tianjin University.

### Tasks

Almost all existing studies manipulated the MWL levels through controlling the task difficulty levels by means of (i) short-term or working memory load, (ii) the number of subtasks to process, or (iii) the speed at which a task has to be performed (Roy et al., 2016b). Verbal N-back and the MATB task with different difficulty levels (easy and hard) were employed in this study. There were two blocks for each condition (eight blocks in total), and each block lasted for 10 min. The participants performed all the blocks in random order and rested for several minutes during the inter-block intervals. In order to reduce the learning effect, the participants were trained with N-back and MATB until their performance reached stable levels. The participants were asked to rate the Mental Effort Rating Scale (RSME) (Paas, 1992) as the subjective MWL index at the end of each block. The RSME is a unidimensional scale that consists of a line with a length of 150 mm marked with nine “anchor points” and has been widely used to measure subjective MWL. Each of the nine “anchor points” was accompanied by a descriptive discourse indicating the degree of effort. The subjects rated the MWL by marking on one of the nine “anchor points” based on subjective judgment.

In the verbal N-back task, the randomly selected consonants were sequentially presented on a screen by the E-prime software (E-prime Psychology Software Tools Inc., Pittsburgh, United States). Each letter appeared for 0.5 s and then disappeared, with a 2.5-s delay between trials. The participants were asked to remember the letters, compare the current letter with the *n*-th before, and respond to all trials by pressing one key for matches and another key for mismatches within the duration between the onset of the current letter and the next one. The proportion of matched trials and mismatched trials was equal. In this study, *n* = 1 and *n* = 3 were, respectively, used in the easy and hard conditions.

The MATB was developed by NASA for performance and workload studies in the laboratory. It simulates the tasks performed by pilots in flight, including a tracking task (TRA), a system monitoring task (SYSM), a resource management task (RESM), and a communication task. In this study, the participants were instructed to perform three tasks (TRA, SYSM, and RESM) concurrently in a continually changing task environment. The communication task was not employed because it depends mainly on auditory function and may interfere with the auditory probes. The two overall MATB difficulty levels (easy and hard) were obtained by manipulating the parameters of the three subtasks (as shown in Table 2) according to the manual of MATB.

**TABLE 2:** Task parameters for multi-attribute task battery.

Overall difficulty	System monitoring task (events/minute)	Resource management task (events/minute)	Tracking task difficulty
Easy	2	1	Low
Hard	20	3.5	High

### Auditory Probes

According to the study of Dyke et al. (2015) complex auditory stimuli were significantly more effective in indexing MWL. Concurrently with performing the tasks, the participants were probed with novel complex sounds (e.g., a door knock, a dog bark, a whistle) randomly selected from a large collection (Fabiani et al., 1996). All sounds were presented at the sound pressure level of 70–90 dB with two speakers placed 80 cm in front of the participants and limited to durations of 350 ms. In order to reduce the “habituation effect” (Strüber and Polich, 2002; Dyke et al., 2015), long intertrial intervals (ITI) were employed and randomly varied between 8 and 22 s. All the participants were instructed to concentrate on the tasks and disregard the auditory probes without any response.

### Electroencephalography Recording and Processing

Sixty-channel scalp EEG data were recorded using NeuroScan SynAmps^2^ and QuickCap with an extended international 10–10 system. EEG data were online referenced to the left mastoid, sampled at 500 Hz, and high-pass-filtered with a cutoff at 0.5 Hz. Then, EEG data were offline re-referenced to the average bilateral mastoid and low-pass-filtered with zero-phase filter cutoff at 45 Hz. The ocular artifacts were removed by independent component analysis after data with large amplitude noises were manually dropped out.

ERP epochs were obtained by extracting the data between 500 ms prior to the auditory stimulus onset and 800 ms post-stimulus. After baseline correction by subtracting the mean value of the pre-stimulus data from each epoch, the epochs were averaged within each participant and each condition to obtain ERPs. A narrow time window around the peak of each component was determined as the ERP component time window in the grand average waveform. Four components were obtained in this study, and their time windows were N1 = 130–170 ms, eP3a = 220–270 ms, lP3a = 280–350 ms, and RON = 390–500 ms. The amplitude of each component was calculated by averaging the amplitudes in the component time window.

In order to compare the cross-task consistency between tir-aERPs and the power spectral density (PSD) of ongoing EEG in indexing MWL, the PSD features were calculated for the 5-s EEG epochs that were extracted from the ongoing EEG between two consecutive ERP epochs using the Welch method with hamming window. Then, the PSDs were averaged within each participant and each condition, and then the averaged PSDs were standardized between 4 and 45 Hz by dividing the total power to obtain the relative PSDs. Based on the findings that the MWL-sensitive PSDs mainly lay in 4–30 Hz, we subsequently analyzed the four bands: θ (4–8 Hz), α (8–13 Hz), β1 (13–20 Hz), and β2 (20–30 Hz). The relative power of each band was determined by the sum of the relative PSDs in the band.

### Discriminant Analysis and Mental Workload Classification

The signed Fisher’s discriminant ratio (*F*_*signed*_) was calculated for each feature of the PSD and ERP as shown in formula (1), where *m*_*E*_ and *m*_*H*_ represent the means of easy and hard conditions of one feature, while σE2 and σH2 represent their variances. The absolute value of *F*_*signed*_ can characterize the discriminant ability between easy and hard conditions, and the sign can tell whether the feature is statistically larger/smaller in the easy condition. To obtain a quantitative measure of cross-task consistency of the discriminant ability that can be compared directly between PSD and ERP features, the absolute value of the difference of *F*_*signed*_ between N-back and MATB [|Δ*F*_signed_|, as defined in formula (2)] was averaged across features separately for PSD and ERP for each subject. A lower mean value of |Δ*F*_signed_| would indicate a higher overall cross-task consistency of the discriminant ability. Because it is hard to exactly match the actual MWL between the two tasks, mismatch MWL may affect the between-task comparisons. The cross-task consistency measure of PSD and ERP should be both affected by the mismatched difficulty, so the results of the comparison of the measures between PSD and ERP should not be affected since the mismatched effect of the difficulty could be balanced in the comparison.


(1) Fs⁢i⁢g⁢n⁢e⁢d=mE-mHσE2+σH2

(2) $$\left|\Delta \,F_{s\,i\,g\,n\,e\,d}\right|=\left|F_{s\,i\,g\,n\,e\,d}^{n-b\,a\,c\,k}-F_{s\,i\,g\,n\,e\,d}^{M\,A\,T\,B}\right|$$

MWL classifications between easy and hard condition were conducted based on PSD and ERP features, respectively, using support vector machine (SVM) with radial basis function kernel as implemented in LIBSVM (Chang and Lin, 2011). Block-wise cross-validations were performed for within-task classification by training and testing SVM with samples from the same task but different blocks. Cross-task classifications were performed in two ways: (i) training SVM on the data from N-back task and testing on the data from MATB (N-back train MATB test) and (ii) training SVM on the data from MATB task and testing on the data from N-back (MATB train N-back test). Due to the low signal-to-noise ratio, the decision values of multiple consecutive ERP trials (*n* = 1, 5, 10) from SVM were averaged to improve the reliability of prediction. This was also done for PSD-based classifications for comparing with ERP. The classification performance was evaluated using the area under the receiver operating characteristic curve (ROC-AUC).

### Statistics

The effects of MWL and task type were examined separately for the amplitudes of tir-aERP components and the relative power of the four frequency bands by performing 1,000-iteration bootstrapping-based non-parametric paired ANOVAs and *t*-tests. Bootstrapping-based *t*-test and ANOVA are distribution independent, more applicable to a small sample size and more accurate than classical parametric methods in practice (Hesterberg et al., 2003). The subjective ratings, the measure of cross-task consistency of the discriminant ability, and the classification performance were compared between PSD and ERP features using parametric bootstrapping-based *t*-tests. The false discovery rate (FDR)-based method (Storey, 2002) was employed to correct the significance level when multiple comparisons were performed.

## Results

### Subjective Ratings

**FIGURE 1:**
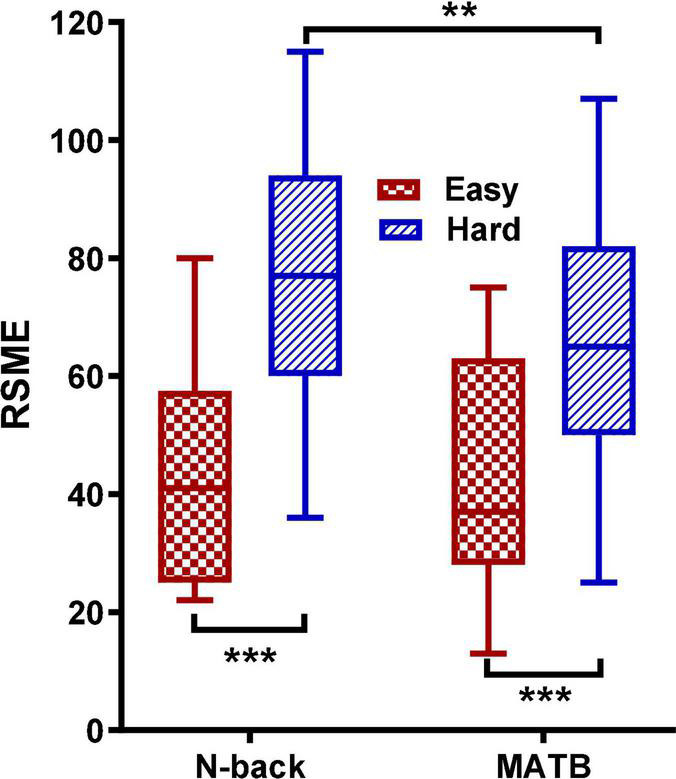
Box plots of Mental Effort Rating Scale for easy and hard conditions in both tasks. The asterisks indicate the significance levels: **p < 0.01; ***p < 0.001.

To ensure that MWL was successfully manipulated, paired *t*-tests were firstly performed to compare the RSMEs (as shown in Figure 1) between easy and hard conditions for both tasks. The results revealed significantly higher RSMEs for the hard condition than the easy in both tasks [N-back: *t*(16) = 5.95, *p* < 0.001; MATB: *t*(16) = 6.47, *p* < 0.001]. No significant difference in RSME was found between the two tasks in the easy condition [*t*(16) = –0.74, *p* > 0.05], but in the hard condition, the RSMEs of N-back were significantly higher than MATB [*t*(16) = 2.98, *p* < 0.01].

### Task-Irrelevant Auditory Event-Related Potentials vs. Power Spectral Densities: General Impressions

**FIGURE 2:**
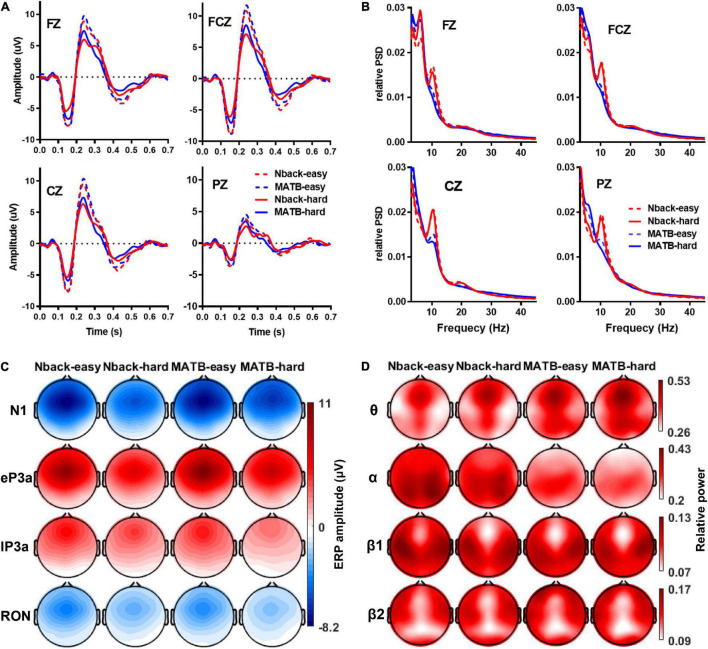
**(A)** Grand average event-related potentials (ERPs) in each condition. **(B)** Grand average relative power spectral density in each condition. **(C)** The topological maps of the grand average ERP amplitude of the four components in each condition. **(D)** The topological map of the grand average relative powers of the four bands in each condition.

Figure 2 shows the curves and topological maps of the grand average ERPs and relative PSDs that can be visually inspected for general impressions of the effects of the task type and MWL. It can be found that the amplitudes of the four ERP components were higher in the easy condition than the hard in both tasks. Although there were some obvious differences between the two tasks, especially for the lP3a, the changes of the ERP amplitudes following MWL were highly consistent across tasks. As for the relative PSDs, there were obvious differences between easy and hard conditions in both tasks; however, obvious differences can also be visually revealed in the MWL-sensitive features between N-back and MATB. The α band that has been found to be sensitive to MWL variation in previous studies seemed to be sensitive to task types in this study. The frontal (FZ) θ power obviously increased in higher MWL in N-back; however, it seemed to change less to MWL variations in MATB.

### Statistical Results for Task-Irrelevant Auditory Event-Related Potentials

**FIGURE 3:**
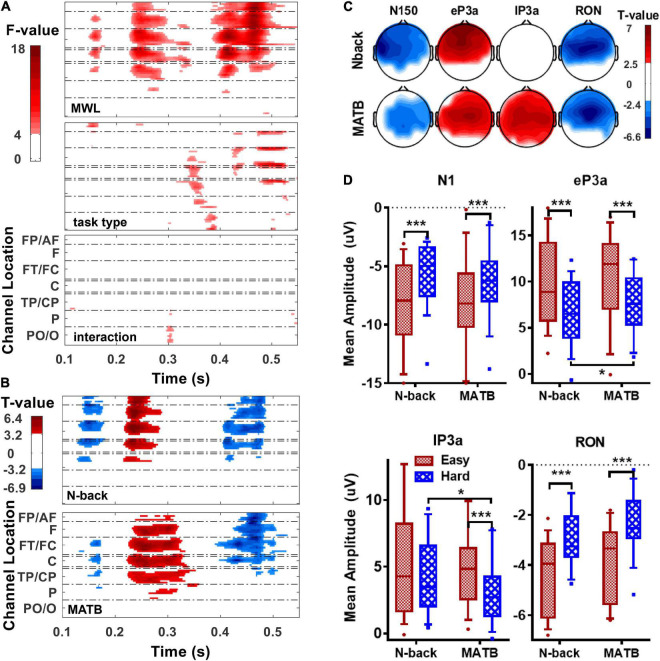
Statistical results for the tir-aERPs. **(A)** The spatial–temporal maps of *F*-values obtained from two-way (MWL × task type) repeated-measure ANOVAs on event-related potentials (ERPs) at each channel and each sample. **(B)** The spatial–temporal maps of *T*-values obtained from paired *t*-tests (easy–hard) on ERPs at each channel and each sample for N-back (upper panel) and multi-attribute task battery (bottom panel). **(C)** The topological maps of *T*-values obtained from paired *t*-tests (easy–hard) on the four ERP components at each channel in N-back (upper panel) and MATB (bottom panel). **(D)** The box plots of the amplitudes and the statistical significance of ERP components at channel Fz. The asterisks in the figure indicate the significance level of the statistical analyses: *p_*FDR*_ <0.05; ***p_*FDR*_ < 0.001. The statistics of no significance (*p*_*FDR*_ > 0.05) were set to 0 in **(A–C)**. AF, anteriofrontal; FP, pre-frontal; F, frontal; FT, frontotemporal; FC, frontocentral; C, central; CP, centroparietal; TP, temporoparietal; PO, parietooccipital; O, occipital.

Statistical analyses were performed on tir-aERPs to reveal the effect for MWL and task type. Firstly, two-way (MWL × task type) repeated-measure ANOVAs were performed on ERP amplitude at each channel and each sample. As shown in Figure 3A, the FDR-corrected results revealed a significant effect for MWL mainly on N1, eP3a, and RON at the anterior regions, a significant effect of task type on lP3a and RON at some sporadic regions, and no significant interaction between MWL and task type. Paired *t*-tests were then performed, respectively, on each sample and each component at each channel to compare between easy and hard conditions in both tasks. According to the results shown in Figures 3B,C, a higher MWL tended to result in lower amplitudes in all the ERP components. Specifically, the component amplitudes of N1, eP3a, and RON were significantly higher in the easy condition than the hard in both tasks, and the significant regions highly overlapped across tasks. However, the amplitudes of lP3a in nearly all regions were only significantly sensitive to MWL in MATB but not N-back. As for channel Fz, the results shown in Figure 3D revealed significantly higher amplitudes of ERP components in the easy condition than the hard for N1, eP3a, and RON in both tasks and lP3a only in MATB (*t* > 3.69, ps_FDR_ < 0.001). Figure 3D also shows the results of comparisons between tasks for the amplitudes of the four components at channel Fz. The results revealed significantly higher eP3a and lower lP3a in MATB than N-back in the hard condition (*t* > 2.25, ps_FDR_ < 0.05) and no significance between tasks in easy condition (ps_FDR_ > 0.05). No significant difference between tasks was found for amplitudes of N1 and RON in both easy and hard conditions.

### Statistical Results for Power Spectral Densities

**FIGURE 4:**
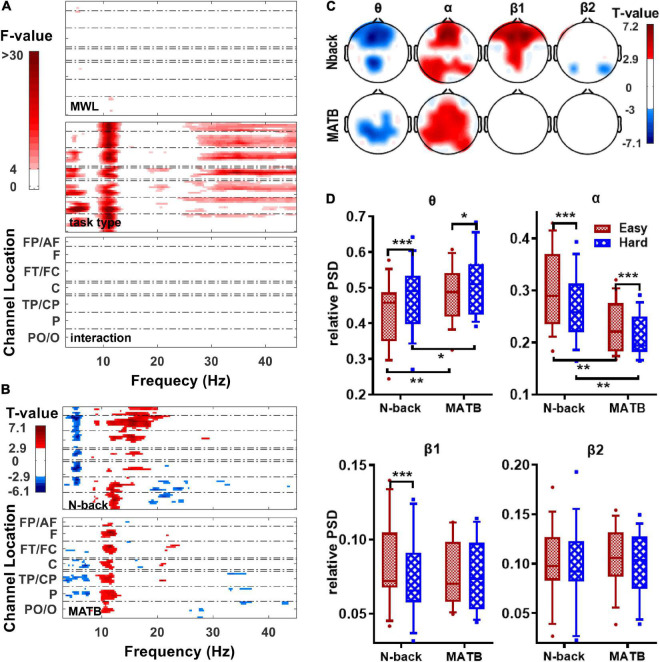
Statistical results for the relative power spectral densities (PSDs). **(A)** The spatial–frequency maps of *F*-values obtained from two-way (MWL × task type) repeated-measure ANOVAs on the relative PSDs at each channel and each frequency. **(B)** The spatial–frequency maps of *T*-values obtained from paired *t*-tests (easy–hard) on relative PSDs at each channel and each frequency in N-back (upper panel) and MATB (bottom panel). **(C)** The topological maps of *T*-values obtained from paired *t*-tests (easy–hard) on the relative powers of the four bands at each channel in N-back (upper panel) and MATB (bottom panel). **(D)** The box plots of the relative PSDs of the four bands at Fz. The asterisks in the figure indicate the significance level of the statistical analyses: *p_*FDR*_ < 0.05; **p_*FDR*_ < 0.01; ***p_*FDR*_ < 0.001. The statistics of no significance (*p*_*FDR*_ > 0.05) were set to 0 in **(A–C)**. AF, anteriofrontal; FP, pre-frontal; F, frontal; FT, frontotemporal; FC, frontocentral; C, central; CP, centroparietal; TP, temporoparietal; PO, parietooccipital; O, occipital.

Figure 4A shows the FDR-corrected results of two-way (MWL × task type) repeated-measure ANOVAs performed on the relative PSDs at each channel and each frequency. The results revealed a significant effect for task type on the relative PSDs of posterior θ, anterior γ, and nearly whole head α but no significant effect for MWL and interaction. The results of paired *t*-tests for N-back task (as shown in Figures 4B,C, upper panel) revealed that the relative powers of θ band at the frontal and parietal regions were significantly lower in the easy condition than the hard and that the relative powers of the mid-frontal and posterior α band and frontal and central β1 band were significantly higher in the easy condition than the hard. As for MATB task, significantly lower parietal θ and higher fronto-central and parietal α relative powers can be revealed from the bottom panels of Figures 4B,C. Specifically, the relative powers at channel Fz were compared between tasks and between conditions using paired *t*-tests. As shown in Figure 4D, besides the differences between easy and hard conditions that have been found above, significantly higher θ power and lower α power in MATB than N-back were found in both easy and hard conditions (*t* > 2.26, ps_FDR_ < 0.05).

### Discriminant Analysis and Classification Results

**FIGURE 5:**
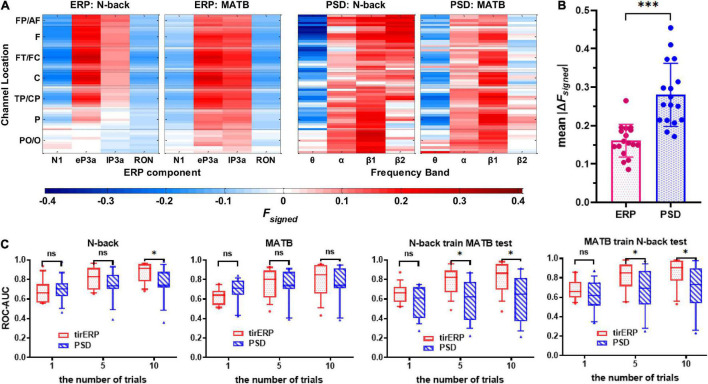
**(A)** Grand average *F*_*signed*_ for each feature of event-related potential (ERP) and power spectral density (PSD). **(B)** Mean values of |Δ*F*_signed_| which were averaged across all features separately for ERP and PSD of each subject. **(C)** Performance (ROC-AUC) for within-task and cross-task classifications separately using PSD and tirERP features. The hashtags and asterisks in the figure indicate the significance level of the paired *t*-tests. ns, no significance; *p_*FDR*_ < 0.05; ***p 0.001.

The grand average *F*_*signed*_ for each feature of ERP and PSD, as shown in Figure 5A, showed a general impression that the discriminant ability of the ERP features was more consistent across tasks than that of PSD features. As a quantitative measure of the cross-task consistency of the discriminant ability that can be compared directly between the ERP and PSD features, the mean |Δ*F*_signed_| of ERP features (0.161 ± 0.043) was significantly lower than that of the PSD features (0.280 ± 0.082) found by paired *t*-test [*t*(16) = 5.809, *p* < 0.001], as shown in Figure 5B.

Figure 5C shows the performance for within-task and cross-task classifications using PSD and tirERP features. It is apparent from the subfigures that the classification performance increased with increasing number of trials. Two-way repeated-measure ANOVAs were performed to examine the effects of the feature (PSD vs. tirERP), the number of trials (1 vs. 5 vs. 10) and their interaction for the within-task and cross-task classification results. As shown in Table 3, no significant effect (*p* > 0.05) of feature was found, but the effects of the number of trials and their interaction were significant (*p* < 0.001) for within-task classifications. As for the cross-task classifications, the effects of feature (*p* < 0.05), the number of trials (*p* < 0.001), and their interactions (*p* < 0.001) were all significant. Paired *t*-tests were then conducted to compare between PSD and ERP features. For within-task results, significantly higher ERP-based ROC-AUC was found only for N-back when 10 trials were used (*p*_*FDR*_ < 0.05), but for the two ways of cross-task classification, the ERP-based results significantly outperformed the PSD-based ones for five and 10 trials (*p* < 0.05) but no significant difference for 1 trial (*p* > 0.05).

**TABLE 3:** Two-way repeated-measure ANOVA results of within-task and cross-task classification results.

	Source	*df*	*F*	Significance
Within task	**N-back**			
	Feature	1, 32	0.87	0.358
	Number of trials	2, 64	28.35	** < 0.0001**
	Feature * number of trials	2, 64	8.42	** < 0.001**
	**MATB**			
	Feature	1, 32	0.612	0.44
	Number of trials	2, 64	51.81	** < 0.0001**
	Feature * number of trials	2, 64	13.32	** < 0.0001**
Cross-task	**N-back train MATB test**			
	Feature	1, 32	5.33	**0.0276**
	Number of trials	2, 64	14.40	** < 0.001**
	Feature * number of trials	2, 64	8.46	** < 0.001**
	**MATB train N-back test**			
	Feature	1, 32	5.60	**0.024**
	Number of trials	2, 64	24.4	** < 0.0001**
	Feature * number of trials	2, 64	9.82	** < 0.001**

*Significant results (p < 0.05) are highlighted in bold.*

## Discussion

The present study firstly investigated the cross-task consistency of the effects of MWL on the tir-aERPs and the ongoing PSDs in the same study framework. To find cross-task-consistent MWL-sensitive EEG features may be an important approach to improve the generalizability of EEG-based MWL estimation methods in different tasks. PSDs have been the most studied EEG features in previous MWL studies; in contrast, the study on tir-aERPs was inadequate. The results of the present study may provide some potential inspirations for revealing higher cross-task-generalizable MWL-sensitive EEG features.

The present study used the novel complex sounds as the auditory probes to evoke robust ERPs and ensure its efficacy in indexing MWL (Dyke et al., 2015). According to the statistical results, the amplitudes of N1, eP3a, and RON decreased under higher MWL in both N-back and MATB tasks. Additionally, the scalp regions in which these components were sensitive to MWL were also highly overlapped in the two different tasks. The most prominent differences between the two tasks were that lP3a significantly decreased with increasing MWL in MATB but not in N-back. The results of the discriminant analyses indicate that the amplitude of eP3a should be most generalizable across tasks in indexing MWL. In contrast, although θ and α powers were MWL sensitive in the same tendency in the two tasks, they were more strongly sensitive to task type. Especially the α band power that has been found to be sensitive to MWL in different tasks in previous studies (Borghini et al., 2014) was very sensitive to task type according to our results.

The similarities and the differences of tir-aERPs and PSDs between tasks in responding to MWL variations should be a problem worthy of a thorough discussion. The two tasks used in this study are highly different: the N-back is a visuo-verbal working memory task that mainly depends on inner attention and working memory resources, while the MATB is a visuo-motor task that demands visual attention and motor responses (Chun et al., 2011; Kiyonaga and Egner, 2013). The MWL of N-back task was manipulated by changing the number of items to be remembered by the subjects, while in MATB task the MWL level was manipulated by changing the number of events and the speed of moving objects that the subjects needed to pay attention and respond to. According to the taxonomy of attention, the N-back task depends mainly on internal attention referring to the selection, modulation, and maintenance of internally generated information, while MATB depends mainly on external attention referring to the selection and modulation of sensory information (Chun et al., 2011; Kiyonaga and Egner, 2013).

The between-task consistency and inconsistency of ERP components in responding to MWL variations can be explained by cognitive resource competition in a dual-task design. Each component of ERPs reflects a cognitive process in the brain, and its amplitude usually depends on the amount of neural (or cognitive) resources employed in the process. According to the cognitive resource theory, the auditory probes were an auxiliary task, and the amount of cognitive resources used to process the auditory probes depends on the cognitive demand of the main tasks, namely, the N-back and the MATB in the present study. Therefore, the differences of tir-aERPs in responding to MWL variations in different tasks may reflect the differences in cognitive resource demand of the main tasks. In this study, the amplitudes of N1, eP3a, and RON decreased in high-load condition in both tasks, and the scalp regions of statistical significance in both tasks were highly consistent. The fact that no prominent effect has been found in between-task comparisons suggests that tir-aERPs may not be sensitive to task type. The most prominent difference of tir-aERPs in responding to MWL between the two tasks was that the amplitude of the lP3a component significantly decreased under high MWL in MATB but not in N-back. Previous studies proposed that eP3a reflects the call for attentional orienting, while lP3a reflects the actual attentional orienting (Čeponienë et al., 2004). A possible explanation may be that lP3a amplitude reflects the amount of external attentional resources involved in processing the auditory probes.

As for the ongoing EEG, the oscillations in different regions reflect the activation or inhibition of neural populations and information transferring. The two tasks used in the present study depend on very different cognitive processing and thus activate or inhibit different neural populations. The differences in frequency bands and brain regions that are sensitive to MWL variations should be a certainty. That should be the reason why the ongoing EEG-based MWL estimation model failed in generalizing across tasks in previous studies (Baldwin and Penaranda, 2012; Ke et al., 2014, 2015a). The current study found a similar tendency in both tasks that θ and α power significantly responded to MWL variations, but the spatial distribution of the regions of significance for θ and α was very different in the two tasks. In the N-back task, the frontal and parietal θ increased with MWL. In the MATB task, θ power also increased with MWL, but it occurred mainly in the central and parietal regions. It has been an often-reported relationship that frontal θ activity increases with memory load in working memory tasks (Jensen and Tesche, 2002; Itthipuripat et al., 2013; Hsieh and Ranganath, 2014; Scharinger et al., 2017). In previous studies, a power increase in the θ band has also been reported at the parietal and central areas in relation to focused attention (Doppelmayr et al., 2008) and demanding or time pressure tasks (Slobounov et al., 2000; Fairclough et al., 2005; Fallahi et al., 2016). When considering the α band, a negative correlation between MWL and α power has been found in the current study as in many previous workload studies (Brouwer et al., 2012; Ke et al., 2015a; Fallahi et al., 2016; Puma et al., 2018; Charles and Nixon, 2019; Tao et al., 2019). The possible explanations for the load effect of α power may have to do with the deactivation of the default mode network (Knyazev et al., 2011; Mo et al., 2013; Bowman et al., 2017) or the activation of the task-related network (Bazanova and Vernon, 2014; Bacigalupo and Luck, 2019). As for between-task comparisons, more valuable results were the significant effects of task type on both θ and α power. The θ power was significantly higher in MATB than in N-back, but it was on the contrary for α power. It should be noted that the scalp regions significantly affected by MWL were very different between the two tasks for both θ and α power. The between-task differences of the ongoing EEG power in responding to MWL variations may be explained by the distinction of neural activation patterns due to different cognitive resources that were mainly involved in performing the two different tasks.

The results of the present study suggest that tir-aERPs should be more generalizable than PSDs in both response tendency and spatial patterns in indexing MWL under different tasks. The results of the discriminant analyses, the lower mean |Δ*F*_signed_| of ERP features, provided direct evidences that the discriminant ability of tir-aERPs was more consistent across tasks. The cross-task classification results that the ERP features outperformed the PSD features further proved the advantages of tir-aERPs in indexing MWL across different tasks. However, the low signal-to-noise ratio (SNR) of ERPs should be a limitation that should be considered in practical applications. According to the ERP-based BCI studies, the average of multi-trial ERPs was usually used to enhance the SNR. This was just the reason why multiple ERP trials have been used to improve the classification results in this study. The temporal resolution may also be a challenge in real-time application because of the long ITI and the low SNR. A possible solution may be to integrate tir-aERPs and ongoing PSDs or other EEG features.

## Conclusion

The generalization of EEG-based MWL estimation across different tasks is important for application in workspace but still a challenging topic. The present study investigated the consistency of EEG features, especially the tir-aERPs and PSDs, in indexing MWL in two different tasks. The results suggested that the amplitudes of tir-aERPs can index MWL more consistently across different tasks compared with the extensively investigated PSD features. Especially the amplitude of eP3a component was negatively correlated to MWL, and the brain regions of significance were highly overlapped in the two tasks. However, the PSD features were significantly affected by task type and showed different spatial patterns in responding to MWL variations in the two tasks. One of the more significant findings to emerge from this study is that the results of the discriminant analyses and classifications provided direct evidences for the significance of tir-aERP features in cross-task MWL classification. These findings suggest the potential of using tir-aERP features to improve the generalization of EEG-based MWL measures and may provide new insights to our understanding of the common neuropsychological essence of MWL across different tasks.

## Data Availability Statement

The raw data supporting the conclusions of this article will be made available by the authors, without undue reservation.

## Ethics Statement

The studies involving human participants were reviewed and approved by the Ethics Committee of Tianjin University. The patients/participants provided their written informed consent to participate in this study.

## Author Contributions

YK and DM designed the study. YK, TJ, and SL collected the data. YK, YC, XJ, and JJ performed the data analyses. YK edited the manuscript. All authors contributed to the article and approved the submitted version.

## Conflict of Interest

The authors declare that the research was conducted in the absence of any commercial or financial relationships that could be construed as a potential conflict of interest.

## Publisher’s Note

All claims expressed in this article are solely those of the authors and do not necessarily represent those of their affiliated organizations, or those of the publisher, the editors and the reviewers. Any product that may be evaluated in this article, or claim that may be made by its manufacturer, is not guaranteed or endorsed by the publisher.
